# Gutta-Percha Cement

**Published:** 1903-06-15

**Authors:** Emil Herbst


### GUTTA-PERCHA CEMENT.

BY EMIL HERBST, D.D.S.

In order to obtain in a single preparation the combined properties of gutta-percha and cement, Dr. Herbst has advised the following method: A given quantity of cement powder is mixed thoroughly with an equal amount of base-plate gutta-percha fillings, and in to the resulting mixture a small amount of cement liquid is incorporated. This paste becomes thoroughly hard and can be advantageously used in setting crowns and bridges. It becomes soft and malleable when heated, and therefore a bridge set with it can be easily removed.

Incidentally he refers to another method of obtaining a gutta-percha-cement compound, which consists in mixing together equal quantities of cement paste and gutta-percha solution. This combination makes a preparation which adheres to the walls of the tooth and becomes slightly soft upon being heated.

Dr. Herbst offers these methods as mere suggestions on the possibilities of mixing cement with gutta-percha with the object of obtaining a material possessing the advantages of both, and he says that the results obtained in the few cases in which he has tried the combination would warrant its further trial by the profession.—*Cosmos.*

